# Complete Genome and Transcriptomes of *Streptococcus parasanguinis* FW213: Phylogenic Relations and Potential Virulence Mechanisms

**DOI:** 10.1371/journal.pone.0034769

**Published:** 2012-04-18

**Authors:** Jianing Geng, Cheng-Hsun Chiu, Petrus Tang, Yaping Chen, Hui-Ru Shieh, Songnian Hu, Yi-Ywan M. Chen

**Affiliations:** 1 Key Laboratory of Genome Sciences and Information, Beijing Institute of Genomics, Chinese Academy of Sciences, Beijing, People's Republic of China; 2 Division of Pediatric Infectious Diseases, Molecular Infectious Disease Research Center, Chang Gung Children's Hospital, Tao-Yuan, Taiwan; 3 Graduate Institute of Basic Medical Sciences, Chang Gung University, Tao-Yuan, Taiwan; 4 Bioinformatics Center, Chang Gung University, Tao-Yuan, Taiwan; 5 Graduate University of the Chinese Academy of Sciences, Beijing, People's Republic of China; 6 Department of Microbiology and Immunology, Chang Gung University, Tao-Yuan, Taiwan; University of Kansas Medical Center, United States of America

## Abstract

*Streptococcus parasanguinis*, a primary colonizer of the tooth surface, is also an opportunistic pathogen for subacute endocarditis. The complete genome of strain FW213 was determined using the traditional shotgun sequencing approach and further refined by the transcriptomes of cells in early exponential and early stationary growth phases in this study. The transcriptomes also discovered 10 transcripts encoding known hypothetical proteins, one pseudogene, five transcripts matched to the Rfam and additional 87 putative small RNAs within the intergenic regions defined by the GLIMMER analysis. The genome contains five acquired genomic islands (GIs) encoding proteins which potentially contribute to the overall pathogenic capacity and fitness of this microbe. The differential expression of the GIs and various open reading frames outside the GIs at the two growth phases suggested that FW213 possess a range of mechanisms to avoid host immune clearance, to colonize host tissues, to survive within oral biofilms and to overcome various environmental insults. Furthermore, the comparative genome analysis of five *S. parasanguinis* strains indicates that albeit *S. parasanguinis* strains are highly conserved, variations in the genome content exist. These variations may reflect differences in pathogenic potential between the strains.

## Introduction

*Streptococcus parasanguinis* is a member of the viridans streptococci that constitute the major population of the oral microbial ecosystem in human. In its primary niche, the oral cavity, *S. parasanguinis* is one of the early colonizers of the tooth surface. The successful adherence of *S. parasanguinis* can serve as a substratum for the adherence of additional oral bacteria and subsequently develop into a mature biofilm called dental plaque [1], [2]. During oral trauma or surgery, oral streptococci may gain access to the bloodstream and cause transient bacteremia. Furthermore, *S. parasanguinis* and other viridans streptococci are the common causes of native and prosthetic heart valve endocarditis [3], [4]. Thus, the ability to evade host immune clearance is critical for the pathogenesis of *S. parasanguinis*.

Studies on the pathogenic factors of *S. parasanguinis*, in spite of its significance in the oral ecosystem and systemic infection, have been limited to two genetic loci, the *fap1* gene cluster [5]–[8] and the *fimCBA-tpx* operon [9], [10] in the past. The *fap1* gene cluster encodes all proteins that participate in the biogenesis of the long fimbriae which are essential for the adherence of *S. parasanguinis* FW213 to the hydroxyapatite discs and optimal biofilm formation [11]–[13]. FimA, a 36-kDa lipoprotein of the FimCBA Mn^2+^/Zn^2+^ ATP-binding cassette (ABC) transporter, is involved in the metal transport [14] and the development of infective endocarditis [15], [16]. Although the cellular location of the FimA determined by anti-FimA serum is at the tips of the long fimbriae of FW213 [9], the precise role of FimA in the adherence to host cells is yet to be defined.

The genomes of several viridans streptococci have been completed [17]–[21] since the completion of the *Streptococcus mutans* genome in 2002 [22]. The complete genome sequences not only allow for detailed analysis of the phylogenic relationship between species but also provide insights into the biology and pathogenic capacity of the streptococci. However, the validation of the genome annotation generally requires extensive analysis. The recent advances in high-throughput RNA sequencing (RNA-seq) have provided a powerful tool for genomic studies at the overall transcription level [23]. RNA-seq has been successfully used to analyze the transcriptomes in several bacteria [24]–[28], and the unexpected complexity of the gene structure and functional plasticity of RNA elements have been reported [24]. Additionally, RNA-seq analysis is effective in defining the operon structure, refining gene annotation, and discovering new genes and noncoding RNAs [24], [25], [27]. The newly developed Applied Biosystems SOLiD platform allows the cost-effective direct sequencing of the whole transcriptome, and the sequencing coverage of each transcript permits a quantitative comparison of the relative expression levels of interested genes [24]. Here we report the complete genome sequence, which has been refined based on the transcriptomes, of the human isolate *S. parasanguinis* strain FW213. Furthermore, we compare the transcriptomes of cells grown in early exponential and early stationary growth stages at single-nucleotide (nt) resolution by using the SOLiD RNA-seq method. The pH and nutrient availability differ drastically between these two growth phases, thus the results of this study also provide an overview on the physiological activity of these two stages. We propose these differences play an essential role in the survival of *S. parasanguinis* in its natural and alternative niches.

## Results and Discussion

### The general features of the *S. parasanguinis* FW213 genome and its basic transcriptomic structure

**Figure 1:**
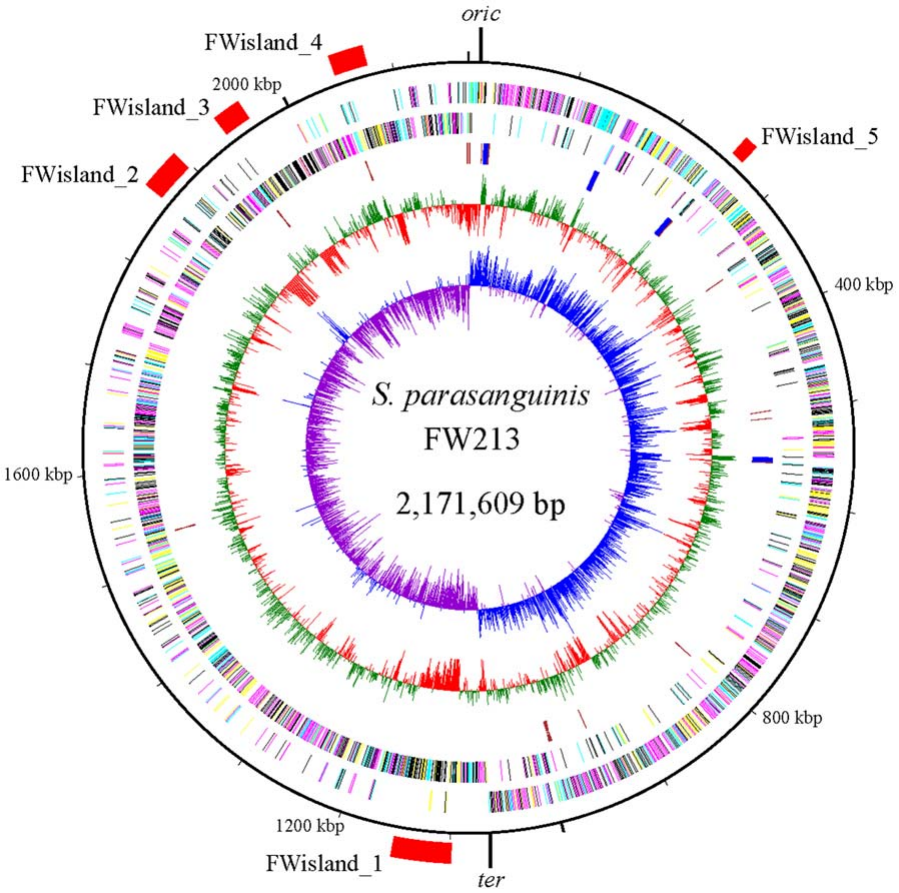
Circular presentation of the *S. parasanguinis* FW213 chromosome. The genome position scaled in kb from base 1 is shown on the outer circle. The second and third circles show the coding sequences on the plus and minus strands, respectively. All genes are color-coded based on the COG functional category as follows: cyan, information storage and processing; yellow, cellular processes and signaling; magenta, metabolism; black, poorly characterized. The fourth circle shows rRNA in cobalt blue and tRNA in brown. The fifth circle shows GC content (in 1-kb windows). The values that are greater than and below the average (41.62%) are in green and red, respectively. The sixth circle shows GC skew curve (10-kb window and 1-kb incremental shift). The values for plus and minus strands are shown in cobalt blue and purple, respectively. The relative locations and sizes of the five putative pathogenic islands are shown outside the scale.

The basic features of the FW213 genome are listed in Fig. 1 and Table 1. This organism also possesses a cryptic plasmid, pFW213. A detailed analysis of pFW213 has been reported previously [29], and will not be discussed in this manuscript. This genome contains 84 hypothetical genes without any matches in the non-redundant protein database; 38 of them are less than 300 bp and are expressed in both the early exponential- and early stationary-phase cultures. As none of these open reading frames (ORFs) matches to the Rfam database [30], these ORFs may encode mini-proteins for various biological processes and regulation in bacteria [31]. 16 transcripts with an average sequence coverage score and a length greater than 100 bp dispersed in the GLIMMER analysis-defined intergenic regions were detected from RNA-seq analysis (Table 1). 10 of these transcripts encode proteins matched to known hypothetical proteins, one is a pseudogene, and the other 5 matched to Rfam database. Additionally, 87 possible small RNA were extracted from intergenic regions by using the integrative computational tool sRNAPredict2 [32] and further confirmed by the transcriptome analysis. Thus the transcriptome not only refines the annotation but also suggests that *S. parasanguinis* utilizes small RNAs to modulate gene expression.

**Table 1:** General features of *S. parasanguinis* FW213 genome.

Category	Characteristics
Genome size (bp)	2,171,609
GC content (%)	41.62
Protein coding	2,019
tRNAs	61
rRNA operons	4
New transcripts^a^	
Match with known protein	10
Pseudogene	1
Match with Rfam database	5
Putative small RNA^b^	87
Plasmid (size in bp)	pFW213 (7078 bp)^c^

a , identified by RNA-seq results.

b , predicted by sRNAPredict2 and confirmed by RNA-seq results.

c , from reference 29.

**Figure 2:**
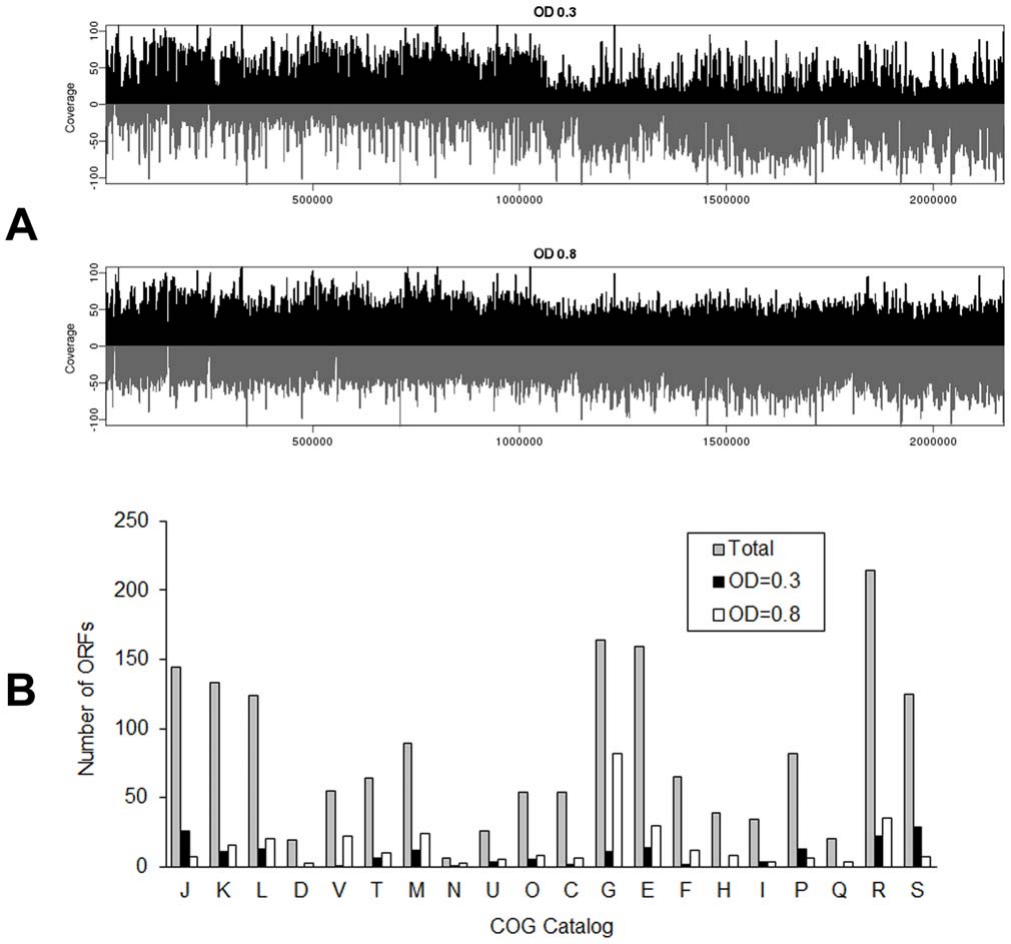
The global transcriptomes of *S. parasanguinis* at OD _600_ = 0.3 and OD _600_ = 0.8. (A) The expression levels of the sense and anti-sense strands are present at single-nt resolution in black and grey, respectively. The coverage is calculated as log(the number of reads+1)/log(1.1). (B) The numbers of genes differentially expressed under the two growth conditions according to COG classification.

Global transcriptomic analyses using RNA-seq confirmed that 1981 and 2007 of 2,020 ORFs were expressed in cultures at OD_600_ = 0.3 and OD_600_ = 0.8, respectively (Fig. 2A and Table S1). The expression levels of 30 randomly selected ORFs were confirmed by RT-PCR (Table S2 and Fig. S1). Among these expressed ORFs, 227 and 395 genes were up-regulated with more than a 2-fold change in RPKM values (*p*<0.05) in cells grown at OD_600_ = 0.3 and at OD_600_ = 0.8, respectively. As expected, most of the genes that were up-regulated in the active growth phase (OD_600_ = 0.3) belong to categories J (translation, ribosomal structure and biogenesis) or L (replication, recombination and repair) of the Cluster of Orthologous Groups (COG), whereas genes in categories G, E, and F that encode proteins for carbohydrate, amino acid (aa) and nt uptake and metabolism were up-regulated when approaching nutrient starvation (Fig. 2B). Similar results have been interpreted from *E. coli* transcriptomic analyses [33], [34]. Of note, 28 ORFs without a predicted function (category S) are up-regulated in the early exponential growth phase. The functions of these ORFs remain to be determined.

**Figure 3:**
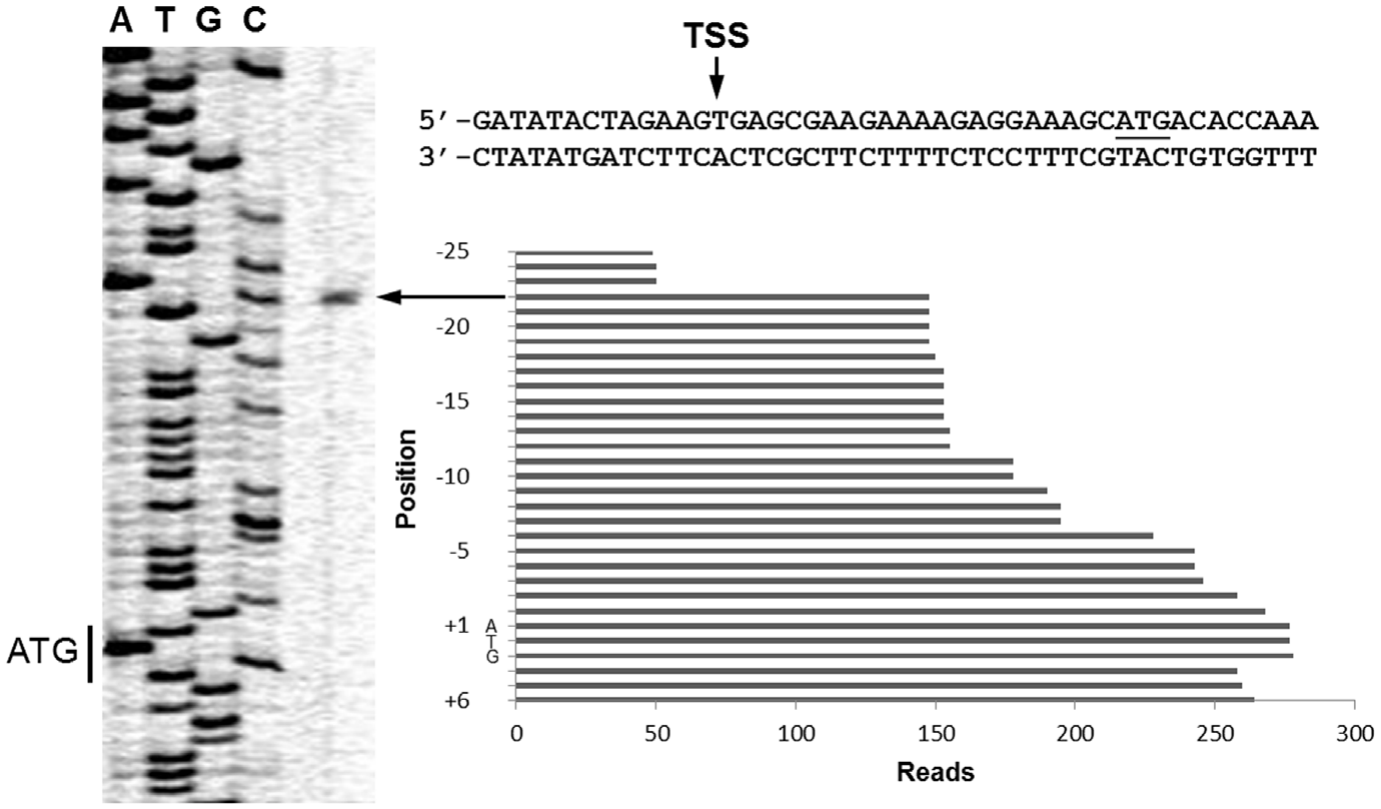
Determination of the transcription initiation site of Spaf_0344. The primer extension analysis of Spaf_0344 is shown on the left side of the figure. The nucleotide sequence of the 5′ flanking region and the read counts at each nucleotide are shown on the right side of the figure. The primer extension analysis was performed with the total cellular RNA of *S. parasanguinis* FW213and a primer located 116-base 3′ to the ATG and containing the antisense sequence of Spaf_0344. The extended product was analyzed alongside a DNA-sequencing reaction by using the same primer. The location of the ATG start site of orf344 and the transcription initiation site (TSS) are indicated.

**Figure 4:**
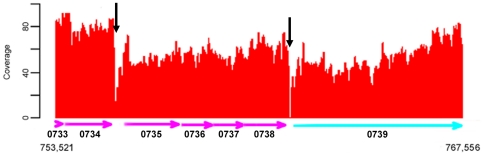
Identification of operons based on the expression profiles. The sequence coverage from 753,521 to 767,556 bp of the *S. parasanguinis* FW213 chromosome is shown. The operon is defined based on continuous expression and sequence coverage change, and the limits of the operon (Spaf_0735-Spaf_0738) are indicated by vertical arrows. The tag number of each gene is listed below the arrows. The ORFs are color-coded as described in Fig. 1 legend. The coverage is calculated as described in Fig. 2 legend.

Attempts were made to determine the transcription initiation of an ORF based on the transcriptome. As an example, the transcription initiation site of Spaf_0344 was mapped to an A located at 22-base 5′ to the translation start site by primer extension analysis (Fig. 3). This result is in agreement with the transcriptomics analysis. A similar result was also observed with the *fimCBA* operon (data not shown). However, the initiation site for *pepO* predicted by the transcriptome was closer to the ATG start codon than the previous determination by primer extension analysis [14]. Previous analysis indicates that *pepO* transcribes from 3 sites, located at 267-, 155-, and 123-base 5′ to the translation start site, respectively. It is likely that the short half-life of the 5′ long untranslated region leads to the discrepancy between these two results. Similarly, we failed to map the end of the transcripts with confidence, presumably due to a high frequency of degradation. Based on the contiguous sequence coverage obtained from RNA-seq analysis, the operon boundaries were also determined through sharp sequence coverage changes (Fig. 4), and further confirmed by RT-PCR. The overall results suggest that there are a total of 427 polycistronic operons and 271 monocistronic genes in FW213. Moreover, different operon organizations within the same region were detected in cells in different growth phases. For instance, Spaf_0314, Spaf_0379, Spaf_0702, Spaf_1731, and Spaf_1764 were part of a polycistronic message in cells grown at OD_600_ = 0.8, while at the stage of OD_600_ = 0.3 these genes were not cotranscribed with the 3′ flanking ORF, suggesting the presence of differential expression and/or termination within an operon in response to growth phases. The presence of alternative transcripts has also been reported in *Halobacterium salinarum* and *Mycoplasma pneumoniae* [28], [35]. Taken together, these findings indicate that transcription regulation in prokaryotes is more complicated than previously thought.

### The comparative genomic analysis of *S. parasanguinis* FW213 with other streptococci

Comparative genomic analysis with *S. mutans* UA159 (AE014133) [17], *Streptococcus pneumoniae* CGSP14 (CP001033) [19], *Streptococcus sanguinis* SK36 (CP000387) [21], *Streptococcus thermophilus* CNRZ1066 (CP000024) [18], *Streptococcus gordonii* CH1 (CP000725) [20], *Streptococcus pyogenes* M1 GAS (AE004092), and *Streptococcus suis* 05ZYH33 (CP000407) revealed that *S. parasanguinis* is most closely related to *S. sanguinis*, although large-scale rearrangements are observed between these two genomes (Fig. S2). Analysis of the orthologous genes also indicated that *S. parasanguinis* is more closely related to *S. gordonii* and *S. sanguinis* than to the other 5 species, consistent with the result of 16S rRNA-based phylogenetic analysis [36]. Furthermore, the genes of *S. parasanguinis* that are without an ortholog in *S. gordonii* or *S. sanguinis* are clustered in 3 acquired-DNA segments (Fwisland_1, Fwisland_2, and Fwisland_4). Interestingly, the 4 acquired-DNA segments (FWisland_1 to FWisland_4) reside in the same replichore (Fig. 1), which could result in lopsided genome architecture across the replication axis. The uneven distribution of the FW213 genome could lead to chromosomal inversion and translocation for stabilizing genome architecture as seen in *S. pyogenes* strain M3 [37].

**Figure 5:**
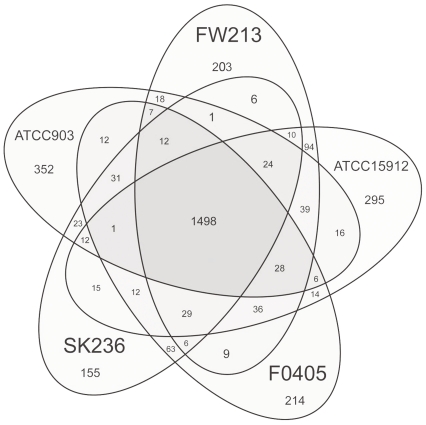
The whole genome comparison between *S. parasanguinis* strains. Venn diagram representation of unique and shared gene numbers between and among strains FW213, ATCC15912, F0405 and SK236.

**Figure 6:**
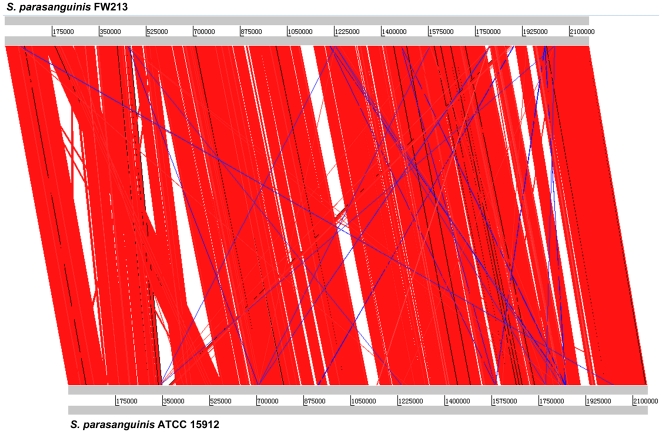
ACT visualization of the whole genome comparison based on BLAST between strains FW213 and ATCC15912. The red and blue bars represent the forward and reverse matches, respectively.

The comparative analysis of the FW213 genome with the complete genome of *S. parasanguinis* ATCC15912 (CP002843), and the drafts of ATCC903 (AEVE00000000), F0405 (AEKM00000000) and SK236 (PRJNA67179) identified a total of 1,498 ORFs that are shared by all 5strains (Fig. 5). In addition to these 1,498 ORFs, FW213 shares 260, 129, 127 and 88 ORFs with strains ATCC15912, ATCC903, F0405 and SK236, respectively, suggesting that FW213 is more closely related to ATCC15912 than to the other strains. Interestingly, a cluster of genes within the proposed FW213 FWisland_1 (see below) is absent in the other 4 genomes. A close examination of the genomes of FW213 and ATCC15912 confirms the rearrangement and variation between these two strains (Fig. 6). Most significantly, both the relative location of *fap1* within FWisland_3 and the deduced aa sequence of Fap1 are different between FW213 and ATCC15912, albeit both proteins contain a serine-rich motif and are of compatible sizes, suggesting that variations in the genomes, perhaps also in the pathogenic capacity, exists between *S. parasanguinis* strains.

### Competence for horizontal gene transfer (HGT)

In contrast to *S. sanguinis* SK36, in which only 2 functional insertion sequence (IS) elements are found, there are 6 copies of IS*111A*, 4 copies of IS*200* family transposases and 19 other transposases in the *S. parasanguinis* FW213 genome. Although some of them appear to be remnants without an active function, these sequences could provide sites for homologous recombination in acquisition of novel genes from related organisms via HGT, which is especially significant in a close contact population, such as the oral biofilm. This genome contains10 genes encoding apparent remnants of phage-related proteins, but is without any intact prophages, demonstrating that HGT via phage infection plays a role in the genome evolution. Interestingly, *S. parasanguinis* is not naturally competent for transformation, but 18 competence-specific genes that are found in naturally competent streptococcal species are present in the genome (Table S2). A close examination revealed that *comC*, encoding the competence-stimulating peptide (CSP), and *comAB*, encoding proteins for the secretion and processing of ComC, are absent in this genome. Furthermore, most of these competence-related genes are expressed at relatively low levels, which is consistent with the phenotype.

### Genomic islands (GIs)

Based on the GC content, 4 possible GIs termed Fwisland_1 to 4 are identified in the FW213 genome (Fig. 1, Table 2). An additional Fwisland_5 is mined through annotation. Sequence and annotation analyses of these islands revealed that these GIs contain known or putative virulence genes and mobility genes (Tables S3, S4, S5, S6, S7). The expression and potential impact of these islands in the physiology and pathogenesis of *S. parasanguinis* are discussed below.

**Table 2:** Properties of the genomic islands in *S. parasanguinis* FW213.

FWisland	Position (nt)	Locus_tag	GC content (%)	Putative Functions
1	1,098,448–1,148,935	Spaf_1090∼1138	34.86	Salivaricin B and nisin production
2	1,861,779–1,896,405	Spaf_1837∼1873	35.96	Bacteriocin production
3	1,940,974–1,964,809	Spaf_1923∼1935	33.63	Fap1 production and fimbriae maturation
4	2,048,864–2,078,214	Spaf_1996∼2016, Spaf_t58	38.20	EPS production
5	252,266–263,533	Spaf_0242∼0254, Spaf_t35	36.94	*adc* operon

#### i. FWisland_1: the salivaricin B and nisin secretion GI

**Figure 7:**
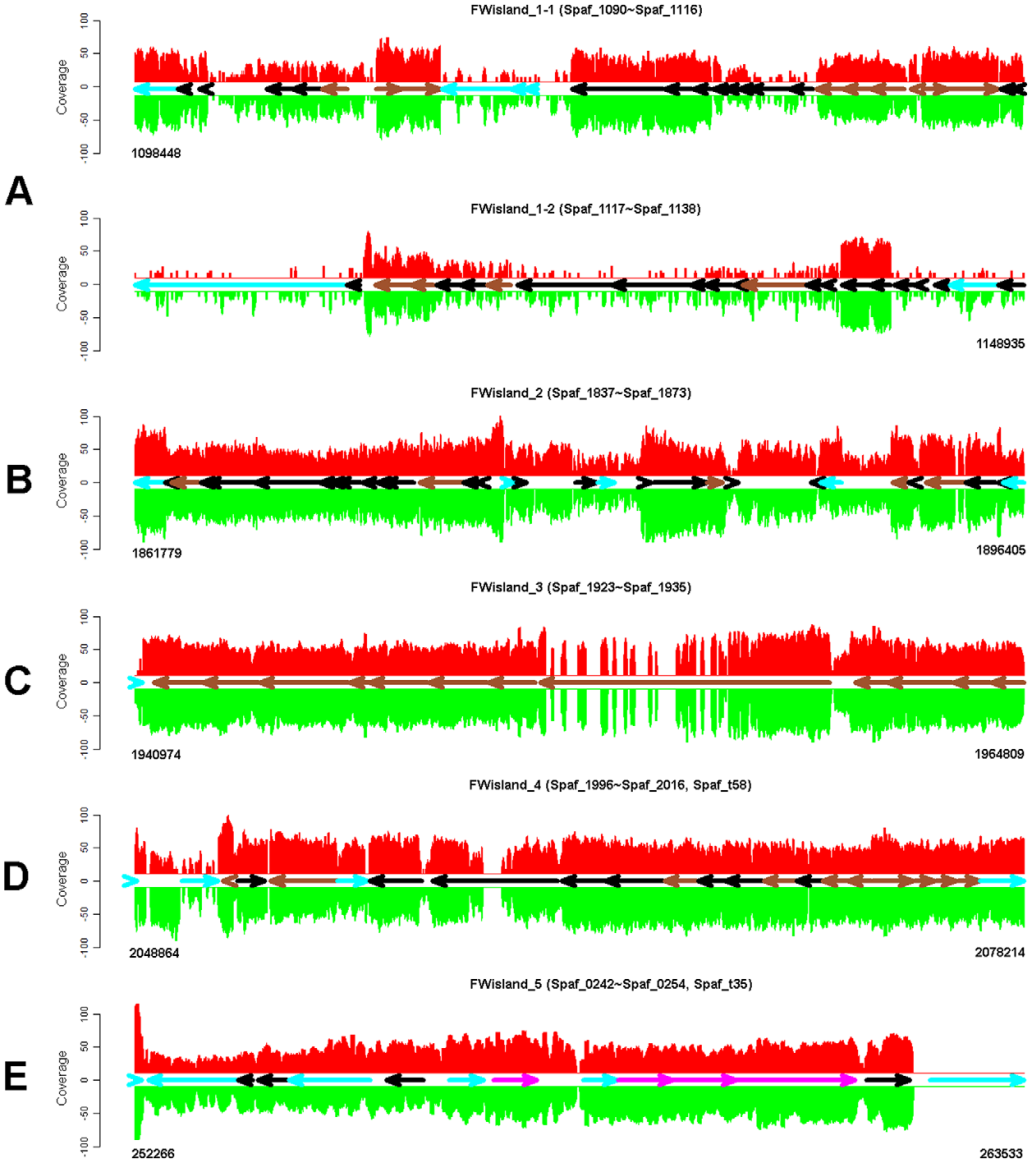
Schematic representation of the putative GIs and their expression in *S. parasanguinis* FW213. (A), FW213island_1; (B), FW213island_2; (C), FW213island_3; (D), FW213island_4; (E), FW213island_5. The expression levels in cultures at OD _600_ = 0.3 and OD _600_ = 0.8 are present at single-nt resolution in red and green, respectively. The relative sizes, locations, and orientations of the ORFs are indicated by color-coded horizontal arrows as described in Fig. 1 legend with the exception that genes of the cellular processes and signaling category are in sienna.

Fwisland_1 contains mosaic mobile elements resembling the conjugative transposon Tn*5253* [38], which is a composite of Tn*5251* and Tn*5252*, with former inserted in the latter (Table S4). Although some of the main components such as *ermAM* and *tetM* of Tn*5253* are absent in FW213, some lantibiotic related genes are included. The first transposon (Spaf_1090 to Spaf_1100 and Spaf_1119 to Spaf_1138) harbors the partial lantibiotic nisin biosynthesis operon [39], indicating that this region has undergone deletion during evolution and *S. parasanguinis* may not produce nisin. The second transposon (Spaf_1101 to Spaf_1118), harbors two operons of the *sboB* locus for the lantibiotic salivaricin B (SboB) production [40]. The first operon (*sboKR*) encodes a putative two component system, and the second operon comprises genes encoding the SboB pre-peptide (encoded by *sboA*) and the immunity proteins (encoded by *sboFEG*). Genes encoding the Tn*5252* relaxase and a putative conjugative transposase are also located within this region. On the other hand, genes encoding proteins for transportation (*sboT*) and modification (*sboM*) of SboB are absent in *S. parasanguinis*. It remains possible that *S. parasanguinis* FW213 modifies and exports SboB by an unknown system other than SboM and SboT. Interestingly, the *sboFEG*, together encoding the subtilin immunity exporter, and the TraG/TraD family protein are also found in this island, which may provide an alternative secretion mechanism for SboB in *S. parasanguinis*. As shown in Fig. 7A, all *sbo* genes were expressed in both growth conditions although the overall coverage of FWisland_1 was generally low, especially in early exponential growth. It has been predicted that the production of bacteria inhibitory substances may provide advantages within a complex ecosystem, such as dental plaque. Thus an up-regulation of this island in the early exponential growth phase may reflect the physiological needs of *S. parasanguinis* in the oral cavity.

#### ii. FWisland_2: the putative bacterocin production GI

This island includes genes encoding proteins for a putative lactococcin production (Table S5). All genes within the island are expressed at both stages of growth (Fig. 7B). Interestingly, the expression of genes encoding the lactococcin 972 type bacteriocin (Spaf_1859) and an ATP-binding cassette (ABC) transporter (Spaf_1860 and Spaf_1861) are up-regulated 3- and 6-fold, respectively, in cells at OD_600_ = 0.8. A similar expression pattern has been observed in the *lclAB* operon of *Lactococcus lactis* PILA972, which encodes the lactococcin 972 and immunity protein [41]. It is tempting to suggest that FW213 also produces a lactococcin-related bacteriocin, and the production is modulated by the growth phase.

#### iii. FWisland_3: the fimbriae encoding and maturation GI

Genes encoding the structure subunit of the long fimbriae, *fap1*, and all enzymes required for the maturation and presentation of the fimbriae are clustered within a 23.9-kp region, designated FWisland_3 (Table S6). Extensive analysis on this island has been previously reported [7], [8]. Homologues of *fap1* and organization of the flanking ORFs are also observed in other oral streptococci such as *S. gordonii* CH1 [20] and *S. sanguinis* SK36 [21], indicating that the biogenesis and glycosylation of the Fap1-like proteins are highly conserved throughout evolution. All genes of this island were expressed at both growth stages (Fig. 7C), and were up-regulated at OD_600_ = 0.8, confirming the impact of the long fimbriae in the biofilm lifestyle [13]. It is also noticed that the expression of *fap1*, encoding the subunit of the long fimbriae, is at relative high levels at both stages, with a 2.8-fold increase at early stationary stage. The up-regulation of FWisland_3 expression and conceivably higher fimbriae presentation shall enhance the biofilm formation of *S. parasanguinis* in the late exponential growth phase, when the biofilm lifestyle may provide better advantages for survival.

#### iv. FWisland_4: the extracellular polysaccharides (EPS) and capsule polysaccharide (CPS) production GI

EPS production plays an essential role in the adherence and initiation of bacterial endocarditis [42], [43]. The EPS production is also associated with endocardial vegetation mediated by many viridans streptococci [44]. FWisland_4 (Table S7) encodes proteins that share strong homology with the protein products of *S. pneumoniae* Type 19F *cpsA-K* genes [45], while only *cpsA*, *cpsB*, *cpsC*, *cpsD*, *cpsE* and *cpsG* are present in *S. sanguinis* and *S. gordonii.* This GI also encodes glycosyltransferases (Spaf_2008 and Spaf_2009) and a putative phosphotransferase (Spaf_2004). Homologs of the *cps19fL*, *cps19fO*, *cps19fN*, and *cps19fM* which are involved in the biosynthesis of dTDP-L-rhamnose in *S. pneumoniae* capsule production are found elsewhere in the FW213 genome (Spaf_1350 to 1352 and Spaf_0821). Together, it is suggested that the products of FWisland_4 participate in the biogenesis and export of EPS and that the repeat unit of polysaccharide structure is similar to that of *S. pneumoniae* type 19F capsular polysaccharide. All genes in this island are expressed at moderate levels at both growth conditions (Fig. 7D), and all EPS production-related genes were up-regulated on average 4-fold in early stationary versus early exponential phase of growth. The polysaccharide capsule constitutes the outermost layer of the cell, and its role in adherence, biofilm formation, and resistant to host phagocytic activity is well documented [46]. The expression of this island may provide basic protection against host immune clearance, and an up-regulation in the late exponential growth phase may further promote the biofilm formation of *S. parasanguinis.*

#### v. FWisland_5: the adc operon

FWisland_5 contains ORFs homologous to known transcriptional regulators, a phosphoglycerate mutase and the *adc* operon consisting of a *adcR*, *adcC*, *adcB*, and *adcA* (Table S8). Gene *adcR* encodes a putative transcriptional repressor for Zn^2+^/Mn^2+^-responsive expression, and *adcCBA* together encode a putative Zn^2+^/Mn^2+^-specific ABC transporter. Furthermore, the histidine-rich metal-binding domain was found in AdcR and AdcA of *S. parasanguinis* FW213. Thus, the *adcRCBA* operon may play an important role in Zn^2+^ and/or Mn^2+^ uptake in *S. parasanguinis* FW213. It is also noticed that the expression of the *adc* operon was up-regulated 3-fold in cells grown at OD_600_ = 0.8 (Fig. 7E), similar to the regulation by AdcR in *S. gordonii* [47]. It was proposed that, in addition to maintaining the intracellular metal homeostasis, AdcR may act as a signal to modulate biofilm formation [48]. As *S. gordonii* and *S. parasanguinis* occupy the same habitat in the oral cavity and both cause subacute endocarditis, it is likely that the Adc system plays a similar role in *S. parasanguinis*.

### Genes encoding proteins that modulate oxidative stress responses, the pathogenicity for endocarditis, host cell lysis, cell wall integrity and osmotic stress responses are induced in the early exponential growth phase

To reach the heart valve successfully and establish infection, *S. parasanguinis* has to evade innate host defenses. An examination of the FW213 genome reveals genes encoding superoxide dismutase (Spaf_0708), thioredoxin (Spaf_0302, Spaf_0423, Spaf_1008 and Spaf_1295), thioredoxin reductase (Spaf_0208 and Spaf_0772), and glutathione peroxidase (Spaf_1379). With the exception of Spaf_0208 and Spaf_0423, all genes were up-regulated in cells at OD_600_ = 0.3 (Table 3). Furthermore, two putative Spx proteins (Spaf_2030 and Spaf_2069), an activator for RNA polymerase under thiol-specific oxidative stress condition [49], were also up-regulated at this stage of growth. Thus, it is possible that the expression of the above genes and the regulation by Spx play a role in early exponential phase of growth.

**Table 3:** Potential virulence factors for the early exponential growth phase in *S. parasanguinis* FW213.

Locus	Annotation	RPKM OD = 0.3^a^	RPKM OD = 0.8^a^	Fold-increase^b^
**I. Oxidative stress responses**				
Spaf_0208	Thioredoxin reductase	511	575	0.9
Spaf_0302	Thioredoxin family protein	188	126	1.5
Spaf_0423	Thioredoxin	105	94	1.1
Spaf_0772	Thioredoxin reductase, putative	480	293	1.6
Spaf_1008	Thioredoxin family protein, putative	330	231	1.4
Spaf_1295	Thioredoxin, putative	1277	551	2.3
Spaf_0708	Mn^2+^-dependent superoxide dismutase	17251	11055	1.6
Spaf_1379	Glutathione peroxidase	342	165	2.1
Spaf_2069	SPX domain-containing protein	6467	1520	4.3
Spaf_2030	Transcriptional regulator Spx	300	42	7.2
**II. Hemolysins**				
Spaf_0610	Hemolysin A, putative	48	67	0.7
Spaf_1208	Hemolysin III-like, putative	121	60	2.0
Spaf_1565	Hemolysin	419	294	1.4
**III. The Fim system**				
Spaf_0347	FimA of FimCBA transporter	408	103	4.0
Spaf_0348	FimB of FimCBA transporter	468	134	3.5
Spaf_0349	FimC of FimCBA transporter	626	185	3.4
**IV. Cell-wall hydrolases**				
Spaf_0018	Putative peptidoglycan hydrolase	4648	2198	2.1
Spaf_1442	Putative 1,4-β-N-acetylmuramidase	2709	823	3.3
Spaf_2091	Transglycosylase	17272	3522	4.9
**V. Osmotic stress responses**				
Spaf_0774	Putative large conductance mechanosensitive channel	10204	3378	3.0
Spaf_1806	Small-conductance mechanosensitive efflux channel	797	240	3.3
Spaf_1558	Putative trehalose-6P hydrolase	848	36	23.4
Spaf_1559	Trehalose-specific IIBC component	449	25	18.1

a , the PRKM was calculated as described in the materials and methods.

b , the ratio of the expression levels in cells grown at OD_600_ = 0.3 divided by that from cells grown at OD_600_ = 0.8.

A greater than 3-fold increase in the expression of *fimCBA* was detected in cells grown at OD_600_ = 0.3 compared to cells of OD_600_ = 0.8 (Table 3). The binding of FW213 to fibrin monolayers via FimA is essential for the development of endocarditis by *S. parasanguinis* [15], and the up-regulation of the *fim* operon at this stage could enhance the colonization of *S. parasanguinis* to the damaged heart valves. It is interesting to note, the expression pattern of the *fim* operon is opposite that of the *adc* operon. Since both the Fim and the Adc systems recognize low and high concentrations of extracellular manganese, respectively, the coordinated regulation of these two systems would ensure an adequate acquisition of essential metal ions for all cell activities.

The annotation also led to the discovery of 3 putative hemolysins (Table 3), two of which (Spaf_1208 and Spaf_1675) are up-regulated in early exponential growth. As the expression of these two proteins is the highest among the three, Spaf_1208 and Spaf_1675 may be associated with the development of bacteremia.

Spaf_0018, Spaf_1442, and Spaf_2091, encoding enzymes catalyze cell-wall digestion, are up-regulated in the early exponential growth phase (Table 3). Spaf_0018 contains a C-terminal DivIC domain required for septum formation and a N-terminal CHAP domain that corresponds to amidase function. Studies on the homologues in *S. pneumoniae* [50] and *S. mutans* [51] indicate that this protein participates in cell-wall biosynthesis and cell division. As this ORF is essential for survival in most bacterial species, functional studies were limited. On the other hand, Spaf_1442 is a putative glycosyl hydrolase for cell wall structure, and studies on the homologues reveal that this ORF mediates both cell-wall metabolism and essential cell activity such as biofilm formation. As demonstrated in *Staphylococcus epidermidis* [52], *Lactococcus lactis* [53] and *S. mutans* [54], the peptidoglycan hydrolase activity is essential for optimal biofilm formation. Thus, the up-regulation of Spaf_1442 in the early exponential growth phase may enhance the initial attachment of *S. parasanguinis* to the tooth surface as well as host tissue. Spaf_2091 is a homolog of IsaA of *Staph. aureus* [55], a suggested soluble lytic transglycosylase. IsaA modulates the overall virulence of *Staph. aureus* by altering the peptidoglycan structure [56]. Spaf_2091 is highly expressed at the early exponential growth phase (Table 3), and thus it is possible that this protein plays a similar role in the early stage of endocarditis infection.

Interestingly, genes encoding the putative conductance mechanosensitive (MS) channels (Spaf_0774 and Spaf_1806) express at relative high levels in the early exponential growth phase, and a 3-fold reduction was detected when cells reached stationary phase. This expression pattern is opposite to the MscS and MscL pattern found in *E. coli* [57], where an up-regulation in the stationary phase is detected. As MS channels are required for the survival of osmotic stress, it is peculiar that *S. parasanguinis* expresses these genes at high levels in the early growth phase. Additionally, a relatively high level of expression was seen in genes encoding the trehalose-specific EIIAB (Spaf_1559) and the trehalose-6 phosphate hydrolase (Spaf_1558) in the early growth phase. Trehalose is a compatible solute that accumulates intracellularly upon osmotic stress [58] and the expression of trehalose PTS in *S. mutans* only occurs in the presence of the substrate [59]. Taken together, systems that are commonly known to participate in osmotic stress may have a very different role in the physiology of *S. parasanguinis.*

### ORFs participate in the acid tolerance response (ATR), alcohol metabolism, extracellular matrix (ECM) binding, and peptide digestion are up-regulated in the early stationary growth phase

ATR is one of the hallmarks for survival in the oral streptococci. A number of mechanisms contributing to the aciduric response have been identified in *S. mutans* [60], [61]. Genes encoding a functional F-ATPase (H^+^-translocating ATPase), the primary factor for maintaining cytoplasmic pH homeostasis in oral streptococci [60], are arranged as an operon in the FW213 genome (Spaf_0740 to Spaf_0747). The expression of this operon is moderately up-regulated in cells grown at OD_600_ = 0.8. The pH value of *S. parasanguinis* cultures at OD_600_ = 0.3 is around 6.8, whereas cultures at OD_600_ = 0.8 is at 5.5–5.6, thus the up-regulation of the *atp* operon at OD_600_ = 0.8 confirms the role of ATPase in pH homeostasis (Table 4). Furthermore, the *arc* operon encoding the arginine deiminase system (ADS) that provides competitive fitness for survival at sub-lethal acidic pH by concomitant production of NH_3_ and ATP in *Streptococcus rattus* and *S. gordonii* [62], [63] is located in the FW213 genome (Spaf_0712 to Spaf_0718). The expression of ADS in *S. gordonii* is regulated by multiple environmental factors, and the expression is enhanced in the stationary growth phase [64]. Similarly, the expression of the *arc* operon in FW213 was up regulated in cells grown at OD_600_ = 0.8, suggesting that the ADS also participates in the ATR of *S. parasanguinis*.

**Table 4:** Potential virulence factors that are up-regulated in the early stationary phase in *S. parasanguinis* FW213.

Locus	Annotation	RPKM OD = 0.3^a^	RPKM OD = 0.8^a^	Fold-increase^b^
**I. Acid tolerance responses**				
Spaf_0740	Subunit C of ATP synthase F0 sector	343	315	0.9
Spaf_0741	Subunit A of ATP synthase F0 sector	915	1358	1.5
Spaf_0742	Subunit B of ATP synthase F0 sector	532	575	1.1
Spaf_0743	Subunit δ of ATP synthase F1 sector	455	495	1.1
Spaf_0744	Subunit α of ATP synthase F1 sector	650	865	1.3
Spaf_0745	Subunit γ of ATP synthase F1 sector	972	1855	1.9
Spaf_0746	Subunit β of ATP synthase F1 sector	1323	1879	1.4
Spaf_0747	Subunit ε of ATP synthase F1 sector	956	1141	1.2
Spaf_0712	Arginine deiminase	1656	8470	5.1
Spaf_0714	Ornithine carbamoyltransferase	1091	9256	8.5
Spaf_0715	Carbamate kinase	54	749	13.8
Spaf_0716	Arginine-ornithine antiporter	1016	2052	2.0
Spaf_0717	Peptidase	472	810	1.7
**II. Alcohol dehydrogenase (ADH)**				
Spaf_0058	ADH, iron-containing	144	1233	8.6
Spaf_0062	Zn-dependent ADH	19	74	3.9
Spaf_0170	ADH, zinc-containing	80	308	3.8
Spaf_0171	ADH, propanol-preferring, putative	41	138	3.3
Spaf_1781	ADH, iron-containing	7	11	1.5
**III. Extracellular matrix binding proteins**				
Spaf_1409	Fibronectin-binding protein, putative	32	57	1.8
Spaf_0420	Collagen-binding protein, putative	98	249	2.5
Spaf_1570	Collagen-binding protein, putative	94	295	3.1
Spaf_1574	Collagen-binding protein, putative	167	415	2.5
Spaf_1943	Collagen-binding domain containing surface protein	117	238	2.0
**IV. Subtilisin family Serine proteases**				
Spaf_0194	Subtilisin family serine protease	38	87	2.3
Spaf_1710	Cell-wall anchored serine protease	2	29	15.6
Spaf_1711	Cell-wall anchored serine protease	1	13	10.4

a , same as in Table 3.

b , the ratio of the expression levels in cells grown at OD_600_ = 0.8 divided by that from cells grown at OD_600_ = 0.3.

A total of 7 alcohol dehydrogenase (ADH) homologues are identified in the FW213 genome and 5 of them are up-regulated at the early stationary phase (Table 4) whereas the other two (Spaf_0456 and Spaf_1747) are not regulated by growth phases. It is peculiar as only 3 and 4 ADHs are found in the *S. gordonii* CH1 and *S. sanguinis* SK36 genomes, respectively. It has been suggested by Kurkivuori and colleagues that the conversion of ethanol to carcinogenic acetaldehyde by bacterial ADH in the oral cavity may promote the development of oral cancer [65]. Thus, the multiple ADHs and relatively abundant expression, especially at early stationary phase, may contribute to not only energy generation but also the development of other oral diseases.

*S. parasanguinis* FW213 possesses 3 collagen-binding protein (CBP) homologues (Spaf_0420, Spaf_1570 and Spaf_1943), one fibronectin-binding protein homologue (Spaf_1409), and a collagen-binding domain containing surface protein (Spaf_1943). The expression of all 3 CBP homologues and Spaf_1943 were up-regulated at OD_600_ = 0.8 by more than 2-fold, whereas an 1.8-fold increase in expression was observed with Spaf_1409 at the same growth stage, indicating that FW213 possess a strong affinity for ECM molecules. The function of Spaf_1943 in bacterial autoaggregation and biofilm formation has been demonstrated recently [66], further supporting the pathogenic role of this ORF in both the oral cavity as well as on heart valves.

3 serine protease (Spaf_0194, Spaf_1710 and Spaf_1711) of the Subtilisin family are identified in FW213. Although only low levels of expression were observed with all 3 ORFs, significant induction was detected in the early stationary phase (Table 4). Previous studies indicate that the production of Challisin by *S. gordonii*, a homologue of Subtilisin, can interfere with the colonization of *S. mutans* in a two-species biofilm system by inactivation of *S. mutans* CSP [67]. Thus, the optimal expression of these serine proteases by FW213 in the later stage of growth may provide competitive advantages for the bacteria within the complex oral ecosystem.

### Other potential virulence factors

The genome also revealed potential virulence traits including drug and metal resistance (Table 5). A copy of *aph* (Spaf_0881) and of *aadK* (Spaf_0970) encoding the aminoglycoside phosphotransferase and aminoglycoside adenylyltransferase, respectively, are found in the FW213 genome; the expression of these genes may count for the relative high minimal inhibitory concentrations (MICs) for aminoglycosides in *S. parasanguinis* FW213. ORFs potentially encoding resistance for β-lactam (Spaf_0010) and bacitracin (Spaf_0519) are also found in the genome. Furthermore, outside the GIs described above, the FW213 genome contains 3 genes encoding putative cation-driven multidrug efflux systems and 14 genes encoding putative ABC-type multidrug transporters; all of which are expressed at moderate to high levels (data not shown), indicating that this microbe possess a strong defense system.

**Table 5:** Additional virulence factors.

Locus	Annotation	RPKM OD = 0.3^a^	RPKM OD = 0.8^a^
**I. Putative antibiotic resistance genes**			
Spaf_0010	β-lactamase class A	62	134
Spaf_0519	Bacitracin resistance protein/undecaprenol kinase	650	335
Spaf_0881	Aminoglycoside phosphotransferase	10	26
Spaf_0970	Aminoglycoside adenylyltransferase	92	63
**II. Metal resistance systems**			
Spaf_0449^b^	Cadmium resistance transporter	90	71
Spaf_0788	Co/Zn/Cd resistance cation efflux protein	298	140
Spaf_1749	Co/Zn/Cd efflux system component	91	52
**III. Other virulence factors**			
Spaf_1788	Pyruvate oxidase	849	4964
Spaf_1798	Peptide methionine sulfoxide reductase	273	436

a , same as in Table 3.

b , Spaf_449, encoding the structure protein (CadD) is part of a 2-gene operon.

A *cadDX* cassette (Spaf_0449 and Spaf_0450) that confers resistance to cadmium and zinc in *S. salivarius* 57.I [68] is also present in the FW213 genome, suggesting that FW213 possesses relatively high MICs for these two metals. Furthermore, both ORFs Spaf_0788 and Spaf_1749 share significant homologies with the cation efflux proteins for cobalt-zinc-cadmium resistance, and both are highly activated in the late exponential growth phase (Table 5). Together, it is predicted that FW213 is relatively resistant to heavy metal killing.

The study by Vriesema and colleagues suggests that the expression of *msrA* [69], encoding methionine sulfoxide reductase, modulates the virulence potential of *S. gordonii* CH1 in the development of endocarditis by enhancing the growth and oxidative stress capacity. The *msrA* homologues (Spaf_1798) in FW213 expressed well at both growth conditions (Table 5). Whether this ORF also plays a similar role in the disease development requires further analysis. On the other hand, the expression of Spaf_1788, encoding pyruvate oxidase for H_2_O_2_ production under aerobic growth [70], was highly activated at the early stationary phase. It has been demonstrated that *S. sanguinis* and *S. gordonii* compete effectively against *S. mutans* by H_2_O_2_ production [70]. Thus, this locus is likely to provide a similar advantage for FW213 within oral biofilm.

### Conclusions

The genome and expression analysis of *S. parasanguinis* FW213 provide basic information on the physiology and potential pathogenic capacity of this bacterium. The comparative genomics and phylogenetic analysis indicate that this genome is shaped by chromosomal inversion, recombination and HGT events. All putative virulence genes, both within the GIs and elsewhere on the chromosome equip this microbe to maintain an ecological niche in dental plaque, escape from host defense and establish infection in heart valves. Ultimately, the availability of the complete FW213 genome sequence will facilitate further studies of this pathogen and the development of diagnostics and vaccines.

## Materials and Methods

### Strain and growth conditions

*S. parasanguinis* FW213, an isolate of human dental plaque [71], was chosen for this study for reasons listed below. First, FW213 is a frequent isolate of the dental plaque. Second, the infectivity of FW213 in subacute endocarditis has been well established in an animal model [15]. Third, the Fap1 of FW213 is a model system for studying Gram-positive bacterial protein glycosylation and the role of glycosylation in bacterial pathogenesis. Finally, FW213 possesses a cryptic plasmid that is not reported in other *S. parasanguinis* strains. To prepare total cellular DNA or RNA from *S. parasanguinis* FW213, bacteria were grown in Todd-Hewitt (Difco) broth at 37°C, in a 10% CO_2_ atmosphere. Total cellular DNA was isolated from the mid-exponential (OD_600_ = 0.6) phase culture as described previously [72]. Total cellular RNA was isolated from the early exponential (OD_600_ = 0.3) and early stationary (OD_600_ = 0.8) growth phases as described previously [73].

### Genome sequencing and annotation

Genome sequencing was performed using the whole genome shotgun strategy [74]. Briefly, total cellular DNA was mechanically sheared and end-repaired by using T4 DNA polymerase (NEB). 4 libraries containing sheared DNA fragments of various lengths (1.5 to 2 kb, 2 to 3 kb, 4 to 5 kb, and 6 kb) were constructed in pUC18. The nt sequences of the library inserts were determined by using the ET terminator chemistry on an ABI 3700 sequencer (Applied Biosystems) and a MegaBACE 1000 sequencer (Amersham Bioscience). Sequences were assembled and edited using PHRED, PHRAP and CONSED (http://www.phrap.org/phredphrapconsed.html). Gaps were closed by primer walking, long-distance PCR and optimized multiplex PCR [75]. Sequences of the reads in low quality regions were resequenced to ensure the accuracy. We acquired usable shotgun-sequencing traces with an average length of 529 bp, resulting in an 8.84-fold sequence coverage. The complete genome sequence of *S. parasanguinis* FW213 has been deposited in the GenBank database with the accession number CP003122.

The start point of the FW213 genome base numbering is set at the replication origin (*oriC*) which is identified by the GC-skew analysis and Ori-Finder software [76]. ORFs were predicted initially with GLIMMER 2.0 [77] at the default settings with a cutoff at 90 nt. Predicted ORFs were validated with translational start codon assignment based on protein homology and ribosomal binding motifs [78]. The deduced aa sequence of each ORF was then BLASTP searched against the nonredundant database of GenBank and the “true proteins” (80% overlapping, E_value<1e^−10^) were extracted. The remaining ORFs and intergenic sequences were BLASTX searched against the nonredundant database and “true ORFs” (the same criteria as above) were identified. The problematic cases such as overlapping proteins were resolved according to the principle described previously [79], [80]. The function of each protein is predicted by searching against the KEGG pathway database [81], the COG database [82] and the InterPro protein family database [83], [84]. Transfer RNAs were predicted with tRNAscan-SE [85], and ribosomal RNAs (rRNAs) were identified based on the similarity to the corresponding genes of other streptococcal genomes. The final annotation was manually inspected by integrating comprehensively the genome annotation and transcriptomic results to further refine the structure of the predicted genes and annotation.

### Comparative genomic analysis

Whole genome sequences alignments of the streptococcal strains were constructed by using the MUMmer package [86]. The orthologs were identified by Inparanoid and MultiParanoid [87]. The ClustalX software [88] was used to align the concatenated sequences from all orthologs. The Artemis Comparison Tool (ACT) [89] was used to view the overall comparison of *S. parasanguinis* FW213 and ATCC15912 genomes.

### SOLiD RNA-seq library construction, sequencing and mapping

The isolated RNA was treated with DNase I and further purified by using RNeasy Kits (Qiagen) to remove residual chromosome. The rRNA was depleted from the sample based on the standard protocols from RiboMius™ Transcriptome Isolation Kits (Invitrogen). The library construction and sequencing were performed followed the standard protocols from SOLiD™ Small RNA Expression Kit (ABI). Only reads with a quality value greater than 8 were selected and used in the mapping. The selected reads were mapped to the *S. parasanguinis* FW213 genome by using the SOLiD™ System Analysis Pipeline Tool (Corona Lite) allowing mismatches up to 5 bases. The first 45 bases of the unmapped reads were then again used in the second-run mapping. This process was repeated one more time with the first 40 bases of the unmapped reads. rRNA reads were filtered prior to the mapping.

### Gene expression analysis

The expression level of a given gene was evaluated by read counts normalized with the total mapped reads and gene length with the RPKM method [90]. The differential expressions of genes between two libraries were analyzed based on the IDEG6 modeling methods [91] and further confirmed by reverse transcription (RT)-PCR. The differentially expressed genes were sorted into 18 cellular functional groups according to the COG database [82]. To determine the transcription initiation site of Spaf_0344, 50 µg of total RNA was hybridized with the IRD-800 labeled primer scaRAS9650 (5′-CATGCGACTGGCGATTTCCTTATTACT) at 42°C for 90 min, followed by RT. The extended products were analyzed alongside a DNA sequencing reaction using the same primer on a 9% gel, and signals were detected on a LI-COR DNA sequencer (model 4000L).

### Operon identification and confirmation

The genome wide strand-specific sequencing coverage was generated with perl scripts based on the results of unique mapping reads. The operon boundaries are defined based on sharp sequencing coverage transitions (greater than a 2-fold difference) between two neighboring genes that are greater than 100 bp apart and are in the same transcription orientation. The adjacent genes located on the complementary strands were considered as members of two operons. The predicted operon boundaries are confirmed by RT-PCR. Specifically, within an operon, the contiguous transcript between two genes with an intergenic region greater than 100 bp was further confirmed by end-point RT-PCR. That two genes with an intergenic region that are less (and equal to) 100 bp and yet were assigned in two separate operons was also checked by RT-PCR.

## Supporting Information

Figure S1 **Comparison of RT-PCR and transcriptome results.** The best-fit line is shown. The two data sets showed a correlation coefficient (*R*) of 0.86.
(TIF)

Figure S2 **The whole genome alignments of** ***S. parasanguinis*** **FW213 (X-axis) with the genomes of 7 streptococcal species (Y-axis), respectively.** Mummer-based genomic display of *S. parasanguinis* FW213 genome pairing with the genome of (A) *S. sanguinis* SK36, (B) *S. gordonii* CH1, (C) S. *pneumoniae* CGSP14, (D) *S. mutans* UA159, (E) *S. pyogenes* M1 GAS, (F) *S. suis* 05ZYH33, and (G) *S. thermophilus* CNRZ1066. The forward matches are displayed in red, and the reverse matches are in cobalt blue.
(TIF)

Table S1 **The RPKM values of all genes in cultures at OD_600_ = 0.3 and OD_600_ = 0.8.**
(XLS)

Table S2 **Confirmation of expression analysis of RNA-seq by RT-PCR.**
(DOC)

Table S3 **The competence-related genes and their expression in** ***S. parasanguinis*** **FW213.**
(DOC)

Table S4 **The features and expression of FWisland_1.**
(DOC)

Table S5 **The features and expression of FWisland_2.**
(DOC)

Table S6 **The features and expression of FWisland_3.**
(DOC)

Table S7 **The features and expression of FWisland_4.**
(DOC)

Table S8 **The features and expression of FWisland_5.**
(DOC)
