# Supplementary figures and images for: Complete Genome and Transcriptomes of Streptococcus parasanguinis FW213: Phylogenic Relations and Potential Virulence Mechanisms
Source: PLoS One. 2012 Apr 18;7(4):e34769. doi: 10.1371/journal.pone.0034769 (PMC3329508; doi:10.1371/journal.pone.0034769)

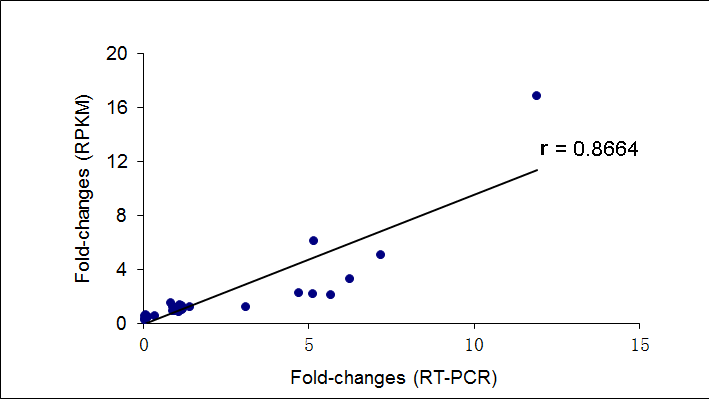

Supplement: Figure S1 — Comparison of RT-PCR and transcriptome results. The best-fit line is shown. The two data sets showed a correlation coefficient (R) of 0.86. (TIF) [file pone.0034769.s001.tif]

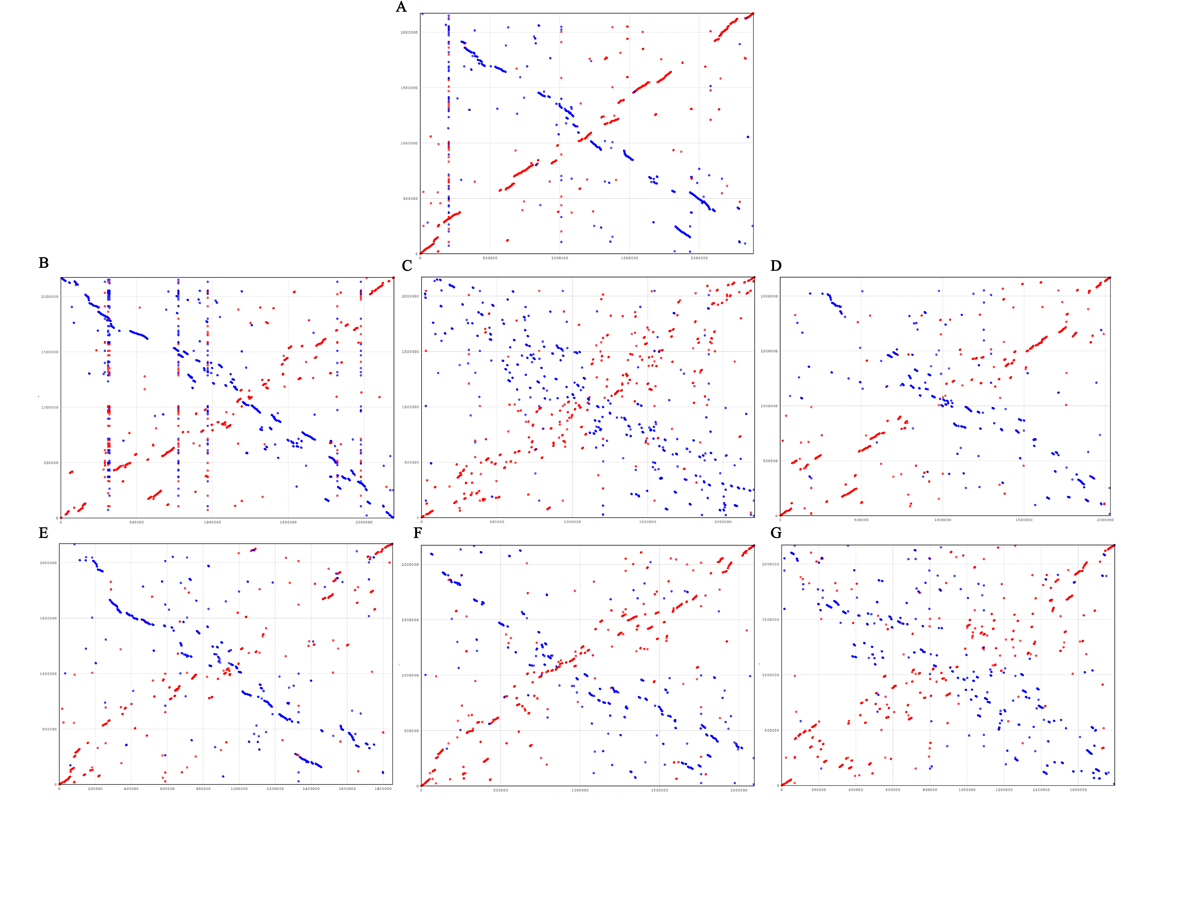

Supplement: Figure S2 — The whole genome alignments of S. parasanguinis FW213 (X-axis) with the genomes of 7 streptococcal species (Y-axis), respectively. Mummer-based genomic display of S. parasanguinis FW213 genome pairing with the genome of (A) S. sanguinis SK36, (B) S. gordonii CH1, (C) S. pneumoniae CGSP14, (D) S. mutans UA159, (E) S. pyogenes M1 GAS, (F) S. suis 05ZYH33, and (G) S. thermophilus CNRZ1066. The forward matches are displayed in red, and the reverse matches are in cobalt blue. (TIF) [file pone.0034769.s002.tif]
