# Supplementary material for: Complete Genome and Transcriptomes of Streptococcus parasanguinis FW213: Phylogenic Relations and Potential Virulence Mechanisms
Source: PLoS One. 2012 Apr 18;7(4):e34769. doi: 10.1371/journal.pone.0034769 (PMC3329508; doi:10.1371/journal.pone.0034769)
Supplement: Table S2 — Confirmation of expression analysis of RNA-seq by RT-PCR. (DOC) [file pone.0034769.s004.doc]

**Table S2. Confirmation of expression analysis of RNA-seq by RT-PCR**

|  | Fold-changes in the expression level^a^ | |
| --- | --- | --- |
| Locus | RPKM | RT-PCR^b^ |
| Spaf_1258 | 0.02 | 0.52±0.09 |
| Spaf_0048 | 0.03 | 0.25±0.05 |
| Spaf_0439 | 0.03 | 0.36±0.04 |
| Spaf_1321 | 0.05 | 0.64±0.09 |
| Spaf_1316 | 0.07 | 0.41±0.11 |
| Spaf_0602 | 0.08 | 0.47±0.12 |
| Spaf_1716 | 0.08 | 0.40±0.12 |
| Spaf_0058 | 0.12 | 0.48±0.08 |
| Spaf_1859 | 0.34 | 0.59±0.09 |
| Spaf_0151 | 0.81 | 1.52±0.01 |
| Spaf_0775 | 0.86 | 1.25±0.1 |
| Spaf_0142 | 0.88 | 0.92±0.02 |
| Spaf_0591 | 0.92 | 0.94±0.03 |
| Spaf_1438 | 1.03 | 1.20±0.05 |
| Spaf_1618 | 1.05 | 0.88±0.14 |
| Spaf_1688 | 1.08 | 1.40±0.25 |
| Spaf_0971 | 1.09 | 1.25±0.17 |
| Spaf_1499 | 1.13 | 1.00±0.09 |
| Spaf_0114 | 1.16 | 1.32±0.06 |
| Spaf_1073 | 1.18 | 1.16±0.36 |
| Spaf_0002 | 1.19 | 1.11±0.06 |
| Spaf_0932 | 1.4 | 1.21±0.03 |
| Spaf_1893 | 3.07 | 1.23±0.16 |
| Spaf_0703 | 4.7 | 2.30±0.27 |
| Spaf_1466 | 5.12 | 2.18±0.26 |
| Spaf_1705 | 5.13 | 6.10±1.31 |
| Spaf_1580 | 5.65 | 2.08±0.14 |
| Spaf_1037 | 6.22 | 3.31±0.92 |
| Spaf_2069 | 7.16 | 5.10±1.90 |
| Spaf_1161 | 11.89 | 16.87±5.38 |

^a^, the ratio of the expression levels in cells grown at OD._600_=0.3 divided by that from cells grown at OD_600_=0.8.

^b^, the numbers are the means and standard deviations of three independent samples.
