# Supplementary material for: Complete Genome and Transcriptomes of Streptococcus parasanguinis FW213: Phylogenic Relations and Potential Virulence Mechanisms
Source: PLoS One. 2012 Apr 18;7(4):e34769. doi: 10.1371/journal.pone.0034769 (PMC3329508; doi:10.1371/journal.pone.0034769)
Supplement: Table S3 — The competence-related genes and their expression in S. parasanguinis FW213. (DOC) [file pone.0034769.s005.doc]

**Table S3. The competence-related genes and their expression in *S. parasanguinis* FW213**

| FW213 locus | RPKM OD=0.3^a^ | RPKM OD=0.8^a^ | Annotation | Homolog in TIGR4^b^ | Homolog in CH1^c^ |
| --- | --- | --- | --- | --- | --- |
| Spaf_0318 | 5 | 171 | ComD histidine kinase | SP_2236 | SSA_2379 |
| Spaf_0317 | 377 | 195 | ComE response regulator | SP_2235 | SSA_2378 |
| None | NA | NA | ComA, permease for competence factor | SP_0042 | SSA_1100 |
| None | NA | NA | ComB, transport protein for competence factor | SP_0043 | None |
| None | NA | NA | ComC, competence stimulating peptide | SP_2237 | SSA_2394 |
| Spaf_0014 | 41 | 10 | ComX, competence-specific σ factor | SP_0014 | SSA_0016 |
| Spaf_0239 | 22 | 24 | ComFA, required for DNA uptake | SP_2208 | SSA_1836 |
| Spaf_0240 | 9 | 16 | ComFC late competence protein | SP_2207 | SSA_1835 |
| Spaf_0285 | 5 | 12 | ComYA late competence gene | SP_2053 | SSA_0184 |
| Spaf_0286 | 3 | 7 | ComYB, DNA transport machinery | SP_2052 | SSA_0185 |
| Spaf_0287 | 1 | 6 | ComYC, translocation protein | SP_2051 | SSA_0186 |
| Spaf_0288 | 2 | 8 | ComYD, DNA transport machinery | SP_2050 | SSA_0187 |
| Spaf_0290 | 1 | 6 | ComGF, DNA transport machinery | SP_2048 | SSA_0189 |
| Spaf_0291 | 2 | 2 | ComGG late competence protein | SP_2047 | SSA_0190 |
| Spaf_0704 | 110 | 29 | ComEA, required for DNA binding and uptake | SP_0954 | SSA_0715 |
| Spaf_0842 | 101 | 99 | ComEB, required for DNA binding | SP_0744 | SSA_1497 |
| Spaf_0705 | 61 | 56 | ComEC, required for DNA binding | SP_0955 | SSA_0716 |
| Spaf_0724 | 0 | 5 | CoiA competence protein | SP_0978 | SSA_0749 |
| Spaf_1071 | 5 | 9 | Smf family DNA processing protein | SP_1266 | SSA_1185 |
| Spaf_0520 | 554 | 351 | MecA negative regulator of competence | SP_1362 | SSA_1958 |
| Spaf_2032 | 99 | 94 | CinA, competence damage-inducible protein | SP_1941 | SSA_2246 |

^a^, the PRKM was calculated as described in the materials and methods. NA, not available.

^b^, the homologs in *S. pneumoniae* TIGR4

^c^, the homologs in *S. gordonii* CH1.
