# Supplementary material for: Complete Genome and Transcriptomes of Streptococcus parasanguinis FW213: Phylogenic Relations and Potential Virulence Mechanisms
Source: PLoS One. 2012 Apr 18;7(4):e34769. doi: 10.1371/journal.pone.0034769 (PMC3329508; doi:10.1371/journal.pone.0034769)
Supplement: Table S4 — The features and expression of FWisland_1. (DOC) [file pone.0034769.s006.doc]

**Table S4**. The features and expression of FWisland_1

| Locus | RPKM OD=0.3^a^ | RPKM OD=0.3^a^ | Annotation | Best BLAST match^b^ | GC Content (%) |
| --- | --- | --- | --- | --- | --- |
| Spaf_1090 | 99 | 147 | Phage integrase | *S. pneumoniae* ATCC 700669 | 30.38 |
| Spaf_1091 | 15 | 30 | Hypothetical protein | *S. pneumoniae* CGSP14 | 29.41 |
| Spaf_1092 | 63 | 127 | Phage associated transcriptional regulator | *S. pneumoniae* G54 | 35.53 |
| Spaf_1093 | 15 | 35 | Hypothetic protein | *Granulicatella elegans* ATCC700633 | 25.88 |
| Spaf_1094 | 11 | 27 | Putative membrane protein | *S. parasanguinis* F0405 | 32.32 |
| Spaf_1095 | 10 | 28 | Bacitracin ABC transporter protein | *G. elegans* ATCC700633 | 34.2 |
| Spaf_1096 | 285 | 287 | NisR | *S. suis* 05ZYH33 | 32.59 |
| Spaf_1097 | 101 | 117 | NisK | *S. suis* 98HAH33 | 28.03 |
| Spaf_1098 | 2 | 14 | Relaxase of Tn*5252* | *S. pneumoniae* CGSP14 | 34.03 |
| Spaf_1099 | 2 | 3 | ORF 9 of Tn*5252* | *S. pneumoniae* CGSP14 | 35.26 |
| Spaf_1100 | 1 | 3 | ORF10 of Tn*5252* | *S. pneumoniae* ATCC 700669 | 33.89 |
| Spaf_1101 | 56 | 172 | recF/RecN/SMC N terminal domain protein | *S. mitis* SK321 | 28.65 |
| Spaf_1102 | 58 | 220 | Signal recognition particle GTPase | *S. pneumoniae* P1031 | 38.21 |
| Spaf_1103 | 37 | 98 | transcriptional regulator | *S. pneumoniae* D39 | 32.7 |
| Spaf_1104 | 3 | 13 | Tn*5253* hypothetical protein | *S. pneumoniae* G54 | 29.26 |
| Spaf_1105 | 4 | 2 | Tn5253 hypothetical protein | *S. pneumoniae* G54 | 33.07 |
| Spaf_1106 | 9 | 2 | Hypothetical protein | *S. pneumoniae* ATCC700669 | 40.69 |
| Spaf_1107 | 1 | 3 | Tn*5253* hypothetical protein | *S. pneumoniae* G54 | 33.7 |
| Spaf_1108 | 1 | 5 | Tn*5253* hypothetical protein | *S. pneumoniae* G54 | 35.52 |
| Spaf_1109 | 31 | 33 | SboG | *S. salivarius* | 33.06 |
| Spaf_1110 | 42 | 46 | SboE | *S. salivarius* | 39.55 |
| Spaf_1111 | 30 | 37 | SboF | *S. salivarius* | 38.5 |
| Spaf_1112 | 31 | 39 | SboA | *S. salivarius* | 35.95 |
| Spaf_1113 | 82 | 100 | SboR | *S. salivarius* | 35.34 |
| Spaf_1114 | 76 | 92 | SboK | *S. salivarius* | 31.14 |
| Spaf_1115 | 18 | 32 | Tn*5253* hypothetical protein | *S. pneumoniae* G54 | 33.99 |
| Spaf_1116 | 39 | 74 | Tn*5253* hypothetical protein | *S. pneumoniae* G54 | 39.01 |
| Spaf_1117 | 0 | 4 | Tn*5253* SNF2-related helicase | *S. pneumoniae* G54 | 36.6 |
| Spaf_1118 | 1 | 4 | Tn*5253* hypothetical protein | *S. pneumoniae* G54 | 38.24 |
| Spaf_1119 | 34 | 51 | NisK | *S. mitis* SK564 | 32.23 |
| Spaf_1120 | 30 | 63 | NisR | *S. mitis* SK564 | 33.33 |
| Spaf_1121 | 8 | 6 | ABC transporter, membrane protein | *S. mitis* SK564 | 31.44 |
| Spaf_1122 | 7 | 4 | ABC transporter, membrane protein | *S. mitis* SK564 | 34.74 |
| Spaf_1123 | 5 | 4 | ATP binding protein of ABC transporter | *S. mitis* SK564 | 36.44 |
| Spaf_1124 | 1 | 5 | Tn*5253* hypothetical protein | *S. pneumoniae* G54 | 38.46 |
| Spaf_1125 | 0 | 4 | Tn*5253* hypothetical protein | *S. pneumoniae* G54 | 37.57 |
| Spaf_1126 | 0 | 7 | Tn*5253* hypothetical protein | *S. pneumoniae* G54 | 34.45 |
| Spaf_1127 | 2 | 8 | Tn*5253* hypothetical protein | *S. pneumoniae* G54 | 38.97 |
| Spaf_1128 | 2 | 4 | Tn*5253* hypothetical protein | *S. pneumoniae* G54 | 44.17 |
| Spaf_1129 | 1 | 13 | Tn*5253* TraG protein | *S. pneumoniae* G54 | 36.58 |
| Spaf_1130 | 3 | 7 | Tn*5253* hypothetical protein | *S. pneumoniae* G54 | 36.88 |
| Spaf_1131 | 4 | 7 | Tn*5253* bacteriocin protein | *S. pneumoniae* G54 | 37.37 |
| Spaf_1132 | 266 | 283 | Hypothetical protein | *S. pneumoniae* ATCC 700669 | 31.41 |
| Spaf_1133 | 215 | 303 | Tn*5253* hypothetical protein | *S. pneumoniae* G54 | 30.84 |
| Spaf_1134 | 0 | 4 | Tn*5253* CAAX N-terminal protease | *S. pneumoniae* G54 | 34.36 |
| Spaf_1135 | 2 | 1 | Tn*5253* hypothetical protein | *S. pneumoniae* G54 | 38.53 |
| Spaf_1136 | 0 | 114 | Tn*5253* hypothetical protein | *S. pneumoniae* G54 | 33.33 |
| Spaf_1137 | 1 | 11 | Tn*5253* DNA methylase | *S. pneumoniae* G54 | 39.1 |
| Spaf_1138 | 0 | 4 | Replication initiator protein A | *S. pneumoniae* Hungary19A-6 | 36.55 |

^a^, the PRKM was calculated as described in the materials and methods.

^b^, the target that the ORF shares the highest homology to.
