# Supplementary material for: Complete Genome and Transcriptomes of Streptococcus parasanguinis FW213: Phylogenic Relations and Potential Virulence Mechanisms
Source: PLoS One. 2012 Apr 18;7(4):e34769. doi: 10.1371/journal.pone.0034769 (PMC3329508; doi:10.1371/journal.pone.0034769)
Supplement: Table S5 — The features and expression of FWisland_2. (DOC) [file pone.0034769.s007.doc]

**Table S5. The features and expression of FWisland_2^a^**

| Locus | RPKM OD=0.3 | RPKM OD=0.3 | Annotation | Best BLAST match | GC Content (%) |
| --- | --- | --- | --- | --- | --- |
| Spaf_1837 | 640 | 731 | Phage integrase family recombinase | *S. parasanguinis* F0405 | 36.03 |
| Spaf_1838 | 39 | 65 | Hypothetical protein | *S. parasanguinis* F0405 | 31.71 |
| Spaf_1839 | 61 | 104 | CHAP domain protein | *S. parasanguinis* F0405 | 41.35 |
| Spaf_1840 | 24 | 48 | Unnamed protein product | *S. thermophilus* | 37.84 |
| Spaf_1841 | 85 | 149 | Conjugal transfer protein | *S. australis* ATCC 700641 | 38.38 |
| Spaf_1842 | 52 | 107 | Conjugal transfer protein | *S. australis* ATCC 700641 | 40.18 |
| Spaf_1843 | 21 | 70 | Conjugal transfer protein | *S. australis* ATCC 700641 | 38.24 |
| Spaf_1844 | 27 | 56 | Conserved hypothetical protein | *S. parasanguinis* F0405 | 45.5 |
| Spaf_1845 | 42 | 98 | Conserved hypothetical protein | *S. parasanguinis* F0405 | 37.87 |
| Spaf_1846 | 59 | 116 | Conjugal transfer protein | *S. infantis* ATCC 700779 | 38 |
| Spaf_1847 | 57 | 75 | Hypothetical protein | *S. parasanguinis* F0405 | 41.56 |
| Spaf_1848 | 114 | 187 | Cro/CI family transcriptional regulator | *S. australis* ATCC 700641 | 40.24 |
| Spaf_1849 | 157 | 282 | FtsK/SpoIIIE family protein | *S. infantis* ATCC 700779 | 38.79 |
| Spaf_1850 | 196 | 199 | Hypothetical protein | *S. infantis* ATCC 700779 | 39.65 |
| Spaf_1851 | 200 | 220 | Hypothetical protein | *S. parasanguinis* ATCC 15912 | 38.78 |
| Spaf_1853 | 83 | 105 | Hypothetical transcriptional regulator | *S. thermophilus* | 32.75 |
| Spaf_1854 | 59 | 73 | pyridoxamine 5'-phosphate oxidase | *S. australis* ATCC 700641 | 29.71 |
| Spaf_1857 | 14 | 18 | Hypothetical protein | *S. parasanguinis* F0405 | 30.89 |
| Spaf_1858 | 15 | 22 | Transcriptional regulator | *S. parasanguinis* F0405 | 35.81 |
| Spaf_1859 | 697 | 2076 | Lactococcin 972 family bacteriocin | *S.* sp. M334 | 36.49 |
| Spaf_1861 | 157 | 1033 | Bacteriocin-associated integral membrane family protein | *S.* *pneumoniae* GA47901 | 28.46 |
| Spaf_1862 | 49 | 630 | Putative bacteriocin ABC transporter | *S. mitis* bv. 2 str. SK95 | 29.7 |
| Spaf_1863 | 3 | 8 | Hypothetical protein | *S. parasanguinis*  ATCC 15912 | 41.86 |
| Spaf_1865 | 32 | 54 | Hypothetical protein | *S. pneumoniae* TIGR4 | 32.14 |
| Spaf_1866 | 290 | 286 | DNA-binding helix-turn-helix protein | *S. oralis* SK255 | 32.52 |
| Spaf_1868 | 488 | 525 | Cell filamentation protein | *S. infantis* SK1302 | 33.66 |
| Spaf_1869 | 201 | 185 | Hypothetical protein | *S. infantis* SK1302 | 30.65 |
| Spaf_1870 | 327 | 365 | ABC transporter | *S. sanguinis* SK115 | 40.16 |
| Spaf_1871 | 167 | 197 | Radical SAM superfamily protein | *S. thermophilus* LMD-9 | 28.87 |
| Spaf_1872 | 568 | 538 | Predicted protein | *S. thermophilus* ND03 | 36.84 |
| Spaf_1873 | 28 | 43 | MutR family transcriptional regulator | *S. thermophilus* CNRZ1066 | 30.19 |

^a^, the footnote is the same as in Table S3.
