# Supplementary material for: Complete Genome and Transcriptomes of Streptococcus parasanguinis FW213: Phylogenic Relations and Potential Virulence Mechanisms
Source: PLoS One. 2012 Apr 18;7(4):e34769. doi: 10.1371/journal.pone.0034769 (PMC3329508; doi:10.1371/journal.pone.0034769)
Supplement: Table S6 — The features and expression of FWisland_3. (DOC) [file pone.0034769.s008.doc]

**Table S6. The features and expression of FWisland_3**

| Locus | RPKM OD=0.3^a^ | RPKM OD=0.8^a^ | Annotation | GC content (%) |
| --- | --- | --- | --- | --- |
| Spaf_1923 | 5 | 90 | Transposase | 38.46 |
| Spaf_1924 | 201 | 485 | Glycosyltransferase, Gtf2 | 32.35 |
| Spaf_1925 | 92 | 321 | Glycosyltransferase, Gtf1 | 32.74 |
| Spaf_1926 | 89 | 318 | Preprotein translocase subunit, SecA2 | 34.46 |
| Spaf_1927 | 118 | 363 | Glycosylation associated protein, Gap3 | 31.64 |
| Spaf_1928 | 100 | 305 | Glycosylation associated protein, Gap2 | 33.4 |
| Spaf_1929 | 96 | 249 | Glycosylation associated protein, Gap1 | 31.68 |
| Spaf_1930 | 180 | 297 | Preprotein translocase subunit, SecY | 30.24 |
| Spaf_1931 | 1165 | 3303 | Fimbriae-associated protein, Fap1 | 38.34 |
| Spaf_1932 | 425 | 862 | Putative glycosyltransferase, GalT2 | 34.58 |
| Spaf_1933 | 128 | 348 | Putative glycosyltransferase, GalT1 | 33.28 |
| Spaf_1934 | 105 | 346 | Nucleotide sugar synthetase-like protein, Nss | 33.23 |
| Spaf_1935 | 109 | 277 | Putative glycosyltransferase, Gly | 32.77 |

^a^, the PRKM was calculated as described in the materials and methods.
