# Supplementary material for: Complete Genome and Transcriptomes of Streptococcus parasanguinis FW213: Phylogenic Relations and Potential Virulence Mechanisms
Source: PLoS One. 2012 Apr 18;7(4):e34769. doi: 10.1371/journal.pone.0034769 (PMC3329508; doi:10.1371/journal.pone.0034769)
Supplement: Table S7 — The features and expression of FWisland_4. (DOC) [file pone.0034769.s009.doc]

**Table S7. The features and expression of FWisland_4^a^**

| Locus | RPKM OD=0.3 | RPKM OD=0.8 | Annotation^b^ | Best BLAST match | GC Content  (%) |
| --- | --- | --- | --- | --- | --- |
| Spaf_t58 | 175 | 165 | tRNA-Cys |  | 53.52 |
| Spaf_1996 | 10 | 22 | Transposase and inactivated derivatives | *S.* *thermophilus* LMD-9 | 37.77 |
| Spaf_1997 | 1455 | 833 | Preprotein translocase subunit SecE | *S.* *sanguinis* SK36 | 34.48 |
| Spaf_1998 | 176 | 237 | Predicted metalloprotease | *S.* *gordonii* CH1 | 49.61 |
| Spaf_1999 | 251 | 129 | Penicillin-binding protein | *S.* *sanguinis* SK36 | 49.55 |
| Spaf_2000 | 91 | 69 | Pseudouridylate synthases | *S.* *sanguinis* SK36 | 47.11 |
| Spaf_2001 | 247 | 221 | R5 protein | S. *agalactiae* 2603V/R | 39.64 |
| Spaf_2002 | 114 | 177 | N-acetylmuramidase/lysin | *S.* *sanguinis* SK36 | 42.79 |
| Spaf_2003 | 229 | 576 | Flippase Wzx, CpsfJ | *S.* *thermophilus* LMD-9 | 31.52 |
| Spaf_2004 | 106 | 467 | Putative phosphotransferase | *S. salivarius* 57.I | 29.31 |
| Spaf_2005 | 107 | 539 | UDP-N-acetylglucosamine 2-epimerase, CpsfK | *S. salivarius* 57.I | 34.81 |
| Spaf_2006 | 55 | 265 | Putative glycosyltransferase, CpsfH | *S. salivarius* M18 | 28.67 |
| Spaf_2007 | 104 | 395 | Oligosaccharide repeat unit polymerase Wzy, CpsfI | *S. salivarius* M18 | 30.09 |
| Spaf_2008 | 97 | 445 | Glycosyltransferase | *S. salivarius* M18 | 31.39 |
| Spaf_2009 | 56 | 306 | Putative glycosyltransferases | *S. salivarius* M18 | 27.45 |
| Spaf_2010 | 51 | 248 | LicD-family phosphotransferase WchP, CpsfG | *S. salivarius* 57.I | 35.16 |
| Spaf_2011 | 52 | 231 | UDP-N-acetyl-D-mannosamine transferase WchO, CpsfF | *S.* *pneumoniae* G54 | 37.55 |
| Spaf_2012 | 243 | 518 | Glycosyl transferase CpsfE | *S.* *sanguinis* SK49 | 43.01 |
| Spaf_2013 | 86 | 230 | Non-specific protein-tyrosine kinase, CpsfD | *S. infantis* ATCC 700779 | 41.3 |
| Spaf_2014 | 121 | 273 | MPA1 family polysaccharide export protein, CpsC | *S.* *gordonii* CH1 | 44.3 |
| Spaf_2015 | 64 | 186 | Wzh/putative phosphotyrosine-protein phosphatase, CpsB | *S.* *gordonii* CH1 | 43.76 |
| Spaf_2016 | 167 | 247 | Capsular polysaccharide biosynthesis protein Cps4A, CpsA | *S. australi*s ATCC 700641 | 42.93 |

^a^, the footnote for RPKM calculation and best Blast match are the same as in Table S3.

^b^, the available homologs in *S. pneumoniae* *cps* locus are listed.
