# Supplementary material for: Complete Genome and Transcriptomes of Streptococcus parasanguinis FW213: Phylogenic Relations and Potential Virulence Mechanisms
Source: PLoS One. 2012 Apr 18;7(4):e34769. doi: 10.1371/journal.pone.0034769 (PMC3329508; doi:10.1371/journal.pone.0034769)
Supplement: Table S8 — The features and expression of FWisland_5. (DOC) [file pone.0034769.s010.doc]

**Table S8. The features and expression of FWisland_5^a^**

| Locus | RPKM OD=0.3 | RPKM OD=0.8 | Annotation | Best BLAST match^b^ | GC content (%) |
| --- | --- | --- | --- | --- | --- |
| Spaf_t35 | 22057 | 16652 | tRNA-Leu | NA | 66.67 |
| Spaf_0242 | 12 | 32 | Integrase | NA | 28.38 |
| Spaf_0243 | 16 | 32 | Hypothetical protein | *S. salivarius* SK126 | 38.02 |
| Spaf_0244 | 37 | 72 | Hypothetical protein | *S. infantis* ATCC700779 | 33.85 |
| Spaf_0245 | 93 | 216 | Tn*916*, transcriptional regulator | *S. agalactiae* 2603V/R | 34.2 |
| Spaf_0246 | 99 | 141 | Hypothetical protein in | *S. agalactiae* 2603V/R | 34.61 |
| Spaf_0247 | 163 | 119 | Transcriptional regulator, Cro/CI family | *S. agalactiae* 515 | 29.56 |
| Spaf_0248 | 291 | 246 | Phosphoglycerate mutase family protein | *Bacillus cereus* | 30.98 |
| Spaf_0249 | 71 | 227 | Putative transcriptional repressor, AdcR | *S. gordonii* CH1 | 38.85 |
| Spaf_0250 | 53 | 213 | ABC transporter, Zn porter, AdcC | *S. sanguinis* SK36 | 43.16 |
| Spaf_0251 | 51 | 154 | ABC transporter, Zn porter, AdcB | *S. sanguinis* SK36 | 44.28 |
| Spaf_0252 | 104 | 264 | Metal-binding (Zn) permease, AdcA | *S. sanguinis* SK36 | 40.45 |
| Spaf_0253 | 202 | 453 | Conserved uncharacterized protein | *S. sanguinis* SK36 | 44.32 |
| Spaf_0254 | 6 | 29 | Transposase, IS*111A*/IS*1328*/IS*1533* | *S. suis* 89/1591 | 39.58 |

^a^, the footnote is the same as in Table S3.

b, NA, not available.
